# The Ecology of New Constituents of the Tick Virome and Their Relevance to Public Health

**DOI:** 10.3390/v11060529

**Published:** 2019-06-07

**Authors:** Kurt J. Vandegrift, Amit Kapoor

**Affiliations:** 1 The Center for Infectious Disease Dynamics, Department of Biology, The Pennsylvania State University, University Park, PA 16802, USA; kurtvandegrift@gmail.com; 2 Center for Vaccines and Immunity, Research Institute at Nationwide Children’s Hospital, Columbus, OH 43205, USA; 3 Department of Pediatrics, Ohio State University, Columbus, OH 43205, USA

**Keywords:** Emerging infectious disease, climate change, flavivirus, Jingmen, Alongshan, *Ixodes*, *Haemaphysalis*, *Dermacentor*, *Ambylomma*, *Hyalomma*

## Abstract

Ticks are vectors of several pathogens that can be transmitted to humans and their geographic ranges are expanding. The exposure of ticks to new hosts in a rapidly changing environment is likely to further increase the prevalence and diversity of tick-borne diseases. Although ticks are known to transmit bacteria and viruses, most studies of tick-borne disease have focused upon Lyme disease, which is caused by infection with *Borrelia burgdorferi*. Until recently, ticks were considered as the vectors of a few viruses that can infect humans and animals, such as Powassan, Tick-Borne Encephalitis and Crimean–Congo hemorrhagic fever viruses. Interestingly, however, several new studies undertaken to reveal the etiology of unknown human febrile illnesses, or to describe the virome of ticks collected in different countries, have uncovered a plethora of novel viruses in ticks. Here, we compared the virome compositions of ticks from different countries and our analysis indicates that the global tick virome is dominated by RNA viruses. Comparative phylogenetic analyses of tick viruses from these different countries reveals distinct geographical clustering of the new tick viruses. Some of these new tick RNA viruses (notably severe fever with thrombocytopenia syndrome virus and Heartland virus) were found to be associated with serious human diseases. Their relevance to public health remains unknown. It is plausible that most of these newly identified tick viruses are of endogenous origin or are restricted in their transmission potential, but the efforts to identify new tick viruses should continue. Indeed, future research aimed at defining the origin, the ecology and the spillover potential of this novel viral biodiversity will be critical to understand the relevance to public health.

## 1. Introduction

Ticks are obligate blood-feeding ectoparasites that belong to the class *Arachnida*, order *Parsitoformes*, and suborder *Metastigmeta* that is further divided into three families, *Ixodidae* (hard ticks), *Argasidae* (soft ticks) and *Nuttalliellidae* [1]. *Ixodidae* is the largest family and it includes four genera and over 700 species of tick which are distributed around the globe [2]. The World Health Organization attributes 17% of the global burden of disease to vector-borne pathogens and ticks are second to only the mosquitoes. The ability of ticks and associated pathogens to spread quickly and threaten public health is illustrated clearly by the recent problems with Lyme Disease in the United States. This disease, which manifests from an infection with the spirochete *Borrelia burgdorferi* [3] was first identified in Connecticut during the investigation of a cluster of infections in school children [4]. Thirty years later, in 2017, 30,000 cases were reported to the Centers for Disease Control and 2018 reports indicate a tripling of overall vector-borne disease, but this increase is primarily driven by ticks [5]. Phylogenetic evidence reveals both the parasite and vector were present long before this recent expansion [6]. This suggests that the recent range expansion of the tick vector and associated disease outbreak is likely related to anthropogenic changes such as reductions in wildlife biodiversity, changing climatic conditions, agricultural intensification and forest fragmentation [7]. These factors are inextricably linked and they can increase interactions between ticks and new hosts, thereby facilitating transmission and the emergence of new pathogens in novel geographic areas [8]. These ongoing dynamics complicate the development of management strategies. Clearly, these efficiently transmitting vectors represent a genuine public health threat and investigations to describe pathogen diversity are warranted.

While Lyme Disease illustrates the ability of tick-borne pathogens to rapidly expand in a dynamic environment, another important tick pathogen, Crimean Congo Hemorrhagic Fever (CCHF), shows the ability of these pathogens to persist endemically across a vast geographic area (30 countries), despite the notable virulence (10–40% mortality) [9]. First described in Crimea, and later linked to similar infections in the Congo, this disease can not only be spread by ticks, but is also transmissible through contact with infectious material or viremic animal tissue. The historic range of this pathogen closely mirrors the range of the main tick vector (*Hyalomma marginatum marginatum*), but recent outbreaks (in Turkey) have been linked to migratory birds dispersing both ticks and pathogens to new areas [10]. So, although CCHF is currently limited to the range of the main vector, other genera and species (including *Ixodes* spp.) of vectors have shown the ability to transmit the virus. Therefore, the potential exists for a pathogen such as CCHF to, like *B. burgdorferi*, expand in geographic distribution rapidly if conditions permit. Overall, the threat posed by ticks and their associated pathogens is great and additional research is needed to better understand issues such as the role of reservoirs, the dynamic ranges of vectors, and the community ecology of the enzootic reservoir and vector cycles. A combination of these factors is what leads to the spillover of pathogens into humans.

Ticks are now well recognized as vectors of several highly pathogenic viruses. However, until a decade ago, only a few tick viruses were known and, among these, flaviviruses were the most well characterized for their geographical prevalence and disease association. Powassan virus is a tick-borne flavivirus present in North America and it is phylogenetically related to the tick-borne encephalitis virus group that was reported from ticks in Europe [11]. Although powassan transmission to humans is rare, the virus is highly pathogenic and often establishes neurotropic infections with fatal consequences [12]. Other flaviviruses of ticks include Kyasanur forest disease virus from the Indian subcontinent, Louping ill virus in Europe and Omsk hemorrhagic fever virus in Asia [13]. Other well-known viruses of ticks include CCHF, which is present in Africa, Asia and Europe, and Colorado tick fever virus, which is present in western United States. Due to a relatively low number of tick-borne virus infections in humans compared to mosquito-borne virus infections, no serious attempts were made to identify new tick viruses until the discovery of Severe Fever with Thrombocytopenia Syndrome virus (SFTSV) from ticks and humans in China in 2011 [14,15]. Soon after, a new pathogenic tick-borne Bunyavirus, named Heartland virus was identified from a patient in the United States [16]. These studies highlight the importance of virus discovery in ticks and, as a result, several investigators have now used metaviromics to identify the virome of ticks in the United States, Europe and more recently, in Australia.

This metaviromics approach has led to the identification of a plethora of new tick viruses [17,18]. These new viruses are highly diverse genetically and often show very distant phylogenetic relatedness to well-characterized human and animal viruses and, therefore, it is difficult to predict the biological properties or host tropism. Additionally, although metaviromics is highly efficient in identifying all viruses in a given sample, the sequencing-based detection of a virus nucleic acid is not generally enough to confirm or refute an ongoing viral infection. The confirmation of an authentic infection requires the isolation of an infectious virus or at least the presence of viral nucleic acids inside the cells. Although our knowledge of viruses in ticks has expanded greatly due to this recent work, follow-up studies to define the origin, ecology and biological properties of these new tick viruses are critical for understanding their relevance to public health. 

## 2. Tick Viruses That Infect Humans and Animals

Most of the well-characterized tick-borne viruses were identified between 1950 and 1975. Long after the first realization that ticks were acting as the vector of pathogenic viruses such as Nairobi sheep disease virus and Louping ill virus in 1931, the first large-scale project to characterize tick viruses was undertaken by the Rockefeller Foundation [19]. In those days, virus discovery relied upon traditional virus isolation in cell culture or via the infection of laboratory mice, followed by virus neutralization assays and analysis of serological cross-reactivity. These efforts led to the identification of approximately 500 new arboviruses, most of these were from mosquitoes, ticks and sand flies. Unfortunately, in the absence of robust DNA sequencing methodologies and even polymerase chain reactions, the complete genome of most of these arbovirus isolates were not sequenced, a requirement for their appropriate phylogenetic classification. Nonetheless, the isolation of infectious tick-borne viruses in cell culture or in animal models not only confirms their authentic infection, but it also reveals their ability to infect mammalian hosts. Here, we are discussing only a few tick-borne viruses that were selected based upon their demonstrated high pathogenicity in humans. 

### 2.1. Powassan and Deer Tick Virus

Powassan virus (POWV) belongs to the family *Flaviviridae* and is serologically related to Tick-Borne Encephalitis Virus complex (TBEV) that also includes Karshi, Kyasanyur forest disease, Langat, Louping ill, Alkhurma, Omsk hemorrhagic fever and Royal Farm viruses. As reflected in the name, POWV was first identified in 1958 in a child with encephalitis from Powassan city in Ontario, Canada [20]. Later, POWV was isolated from *Haemaphysalis longicornis* ticks in Russia in 1972. In 1997, a close genetic relative of POWV was identified in *Ixodes damimni* and was named Deer Tick Virus (DTV) [21]. Phylogenetic analyses of POWV and DTV indicate that these viruses belong to two separate genetic lineages that share about 85% nucleotide and 93% protein identity over their entire genomes. Like other flaviviruses, POWV and DTV are more diverse in structural protein regions, such as the envelope, and are more conserved in non-structural protein regions, such as the helicase and the RNA-dependent RNA polymerase. Although both POWV and DTV are reported from *I. scapularis*, POWV is common in *I. cookei* and *I. marxi* and medium-sized mammals such as *Marmota monax* (Woodchucks), while DTV is common in *I. scapularis* and small mammals, such as White-Footed Mice (*Peromyscus leucopus*) [22]. 

Infection with POWV in humans is mostly reported in the United States and the estimated fatality rate is 10% [12]. The virus appears to be endemic to ticks of the north-east region of the United States and along the Canadian border. Although human infections of POWV and DTV are rare, incidence rates have been increasing recently [12,23]. From 1958 to 1998, only 27 cases of POWV were reported; from 1999 to 2016, in just 7 years, 98 cases of POWV have been reported [22]. Notably, some recent fatal cases were confirmed to be associated with DTV [24,25]. Considering the increasing geographic distributions of *I. scapularis* and *P. leucopus*, it is expected that DTV-associated cases will become more common compared to POWV [22]. 

### 2.2. Crimean–Congo Hemorrhagic Fever Virus

CCHFV belongs to the genus *Orthonairovirus* in the family *Nairoviridae* and is an enveloped virus with a tripartite RNA genome of negative polarity. The main vector of CCFHV is *Hyalomma marginatum*, a tick that is widespread in the regions of Africa, Europe, Asia and the Middle East. The virus was first found in Crimea in 1944 and later in the Congo in 1969 [26]. Infections manifest as acute febrile illness and have a mortality rate up to 40%. Notably, CCHFV transmission can also occur through contact with dead infected ticks, contaminated human blood and other body fluids. So far, thousands of human cases have been reported worldwide, mostly as outbreaks of illness in agricultural workers or in people exposed to the contaminated blood of livestock [26].

Phylogenetic analysis of CCHFV isolates from different countries reveals the existence of at least seven distinct genetic clades, indicating that a tremendous amount of diversity exists [27]. Interestingly, a correlation was observed between the geographical regions and the genetic clades of CCHFV, indicating distinct origins of disease outbreaks from viruses infecting an indigenous population of ticks. Molecular epidemiology of isolates also indicates that, on occasion, infections are caused by the introduction of viruses from genetic clades that are not endemic to the area, suggestive of long-distance dispersals of the vector and the virus [26].

### 2.3. Severe Fever with Thrombocytopenia Syndrome Virus

SFTSV was first isolated in 2009 from patients in China who presented with fever, thrombocytopenia, leukocytopenia, and multi-organ dysfunction [28]. More recently, SFTSV infections have been confirmed in both South Korea and Japan [29]. The virus belongs to the genus *Phlebovirus* of the family *Bunyaviridae*. The initial outbreak consisted of 171 patients from six provinces of China, indicating a widespread infection. After the initial identification of SFTSV from human samples, epidemiological surveillance indicated the presence of this virus in *Haemaphysalis longicornis* ticks. These ticks predominantly feed on farm animals including goats, sheep, cattle, and horses, but they can also be found on companion animals including dogs and cats. Phylogenetic analyses indicate a close genetic relatedness among the SFTV isolates of humans and animals, indicating a shared origin [30]. Notably, and similar to CCHFV, SFTSV it can also transmit between humans through contact with blood or body fluids [31]. 

### 2.4. Colorado Tick Fever Virus

CTFV is an RNA virus with a segmented genome of positive polarity and it belongs to the genus *Coltivirus* in the family *Reoviridae*. The principle vector of CTFV is the Rocky Mountain Wood Tick, *Dermacentor andersoni.* Thus far, human cases of CTFV infection have been reported from Wyoming, Montana, Utah, Oregon, Idaho and Colorado [32]. A recent case of human infection was reported in Alberta, Canada. This virus also manifests as an acute febrile illness, commonly referred to as mountain tick fever. A total of 75 CTFV cases have been identified from 2002 to 2012 [32]. 

### 2.5. Hearthland Virus

Heartland virus is a newly identified phlebovirus that was first isolated from two northwestern Missouri farmers who were hospitalized with fever, leukopenia, and thrombocytopenia in 2009 [16]. Genomes of these viruses are comprised of three segments of single-strand negative-sense RNA that code for the nucleocapsid proteins (segment S), envelope proteins (Segment M) and the polymerase (segment L). The index case had a history of tick exposure and, soon after, the virus was detected in the Lone Star Tick [33] (*Amblyomma americanum*)*,* suggesting a tick-borne virus infection in the index cases. During the period 2012–2013, a total of six cases of heartland virus were identified and all patients were men older than 50 years. Viral surveillance studies indicate the persistent infection of heartland virus in *Amblyomma americanum* ticks as evident by the presence of the virus in ticks collected over several years [34]. Additionally, a recent sero-prevalence study indicates that up to 1% of the normal blood donors have antibodies against the heartland virus, indicating exposure to the virus [35].

## 3. Composition of the Tick Virome

While the traditional approach of virus identification using cell culture or via animal models was very successful, several tick viruses remained unidentified as shown by many recent metaviromics studies [17,36,37,38,39,40,41]. The advancement in high-throughput sequencing technologies in the last 15 years has revolutionized the identification of bacteria, viruses and other microbes [42]. Since viruses do not share a universal anchor sequence, such as a 16S or 28S rRNA gene. An unbiased amplification of all nucleic acids followed by their sequencing (metagenomics or metaviromics) is currently the most appropriate way to identify all the viruses present in a sample. In the following section, we describe new tick viruses and also compare the virome compositions of ticks from different geographical regions, as reported in recent publications [14,16,17,18,32,36,37,38,39,40,41,43].

### 3.1. A Comparison of Tick Viromes from Different Countries

**Figure 1:**
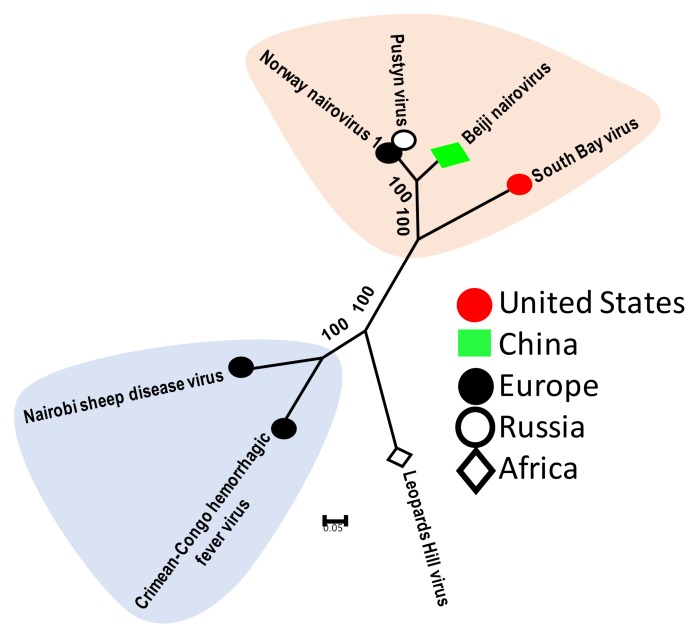
Phylogenetic relatedness of newly identified and known tick nairoviruses. This analysis involved seven amino acid sequences of the viral polymerase gene. Genbank accession numbers of sequences used for phylogenetic analysis are: ASY03236 Norway nairovirus 1, AXQ59276 Beiji nairovirus, AAZ38661 Crimean–Congo hemorrhagic fever virus, YP_009111284 Leopards Hill virus and YP_009361832 Nairobi sheep disease virus.

We compared the metagenomics-based virome of ticks collected from five countries, namely the United States, Norway, France, Australia and China (Table 1) [17,36,37,38,39,41,44]. One of the key features of the global tick virome is the near absence of DNA viruses. Notably, some studies reported the presence of circoviruses, torque viruses and denso-like viruses in ticks. However, the almost universal presence of these viruses in the environmental metagenome casts doubt about an authentic infectious origin in ticks. The global virome of ticks is dominated by RNA viruses that include viruses with single-strand or double-strand, negative- or positive-sense, monopartite or segmented genomes. These data indicate that ticks offer a unique niche for RNA viruses. Among the single-strand RNA viruses of positive-sense genomes, *Flaviviruses* were the most common, followed by viruses belonging to the families *Nodaviridae, Tetraviridae, Picornaviridae, Caulimoviridae, Virgaviridae, Narnaviridae, Luteoviridae* and *Sobemoviridae*. Worldwide, the most common constituents of tick viromes were single-strand negative-sense RNA viruses belonging to the orders *Bunyavirales* and *Monogenavirales* [45,46]. Viruses with monopartite genomes included several genetically diverse viruses belonging to the new virus families *Chuviridae* and *Rhabdoviridae* (Figure 1). Viruses with segmented genomes included members of the families *Phenuviridae*, *Nairoviridae* and *Orthomyxoviridae*. The only double-strand RNA viruses found in ticks belonged to the genus *Colitvirus* of the family *Reoviridae*. A detailed description and comparative phylogenetic analysis of some common and newly identified tick viruses is described in the next section. All evolutionary analyses were conducted in MEGA7 [47]. The evolutionary histories were inferred using the Neighbor-Joining method and evolutionary distances were computed using the p-distance method [48]. All four trees are drawn to scale, with branch lengths in the same units as those of the evolutionary distances used to infer the phylogenetic tree. The rate variation among sites was modeled with a gamma distribution (shape parameter = 1). All ambiguous positions were removed for each sequence pair. All sequences used in phylogenetic analyses were extracted from GenBank and their accession numbers are provided in figure legends. 

### 3.2. Newly Identified Tick Viruses

#### 3.2.1. Tick *Nairoviruses*

These viruses belong to the family *Nairoviridae* of the order *Bunyavirales.* Tick nairoviruses include the well-characterized CCFHV as well as several recently identified viruses such as South Bay Virus (United States), Norway nairovirus and Beiji nairovirus (China). Genomes of these viruses are comprised of three segments of single-strand negative-sense RNA that code for the nucleocapsid proteins (segment S), envelope proteins (Segment M) and the polymerase (segment L), but, to date, only two of the three segments have been identified, namely the S and L segments. Notably, the M (envelope) segment of bunyaviruses is also known to be the most divergent part of the viral genome and, thus, despite being present in the analyzed tick samples, the M segment could have eluded identification due to high genetic diversity (i.e., beyond what is identifiable using sequence-similarity-based algorithms). Among the newly identified tick nairoviruses, South Bay virus (SBV) is the most well characterized. The virus was first identified in *I. scapularis* ticks collected from the United States and was determined to be present in 20% of individual ticks [17]. The sequencing of SBV isolates from different individual ticks indicated high genetic similarity (98–100% nucleotide identity) between the isolates. Similarly, Norway nairovirus-1 and their genetic relative Pustyn virus (Russia) also display close genetic similarity. Although the exact infection prevalence of Norway nairovirus was not determined, the virus was present in five of six tick pools as an abundant virome constituent [38]. Overall, these newly identified tick nairoviruses are closely related and they form a genetic clade that is distinct from CCHFV, Nairobi sheep disease virus, and Leopard Hill virus, the latter isolated from bats (Figure 1).

#### 3.2.2. Tick Phleboviruses

**Figure 2:**
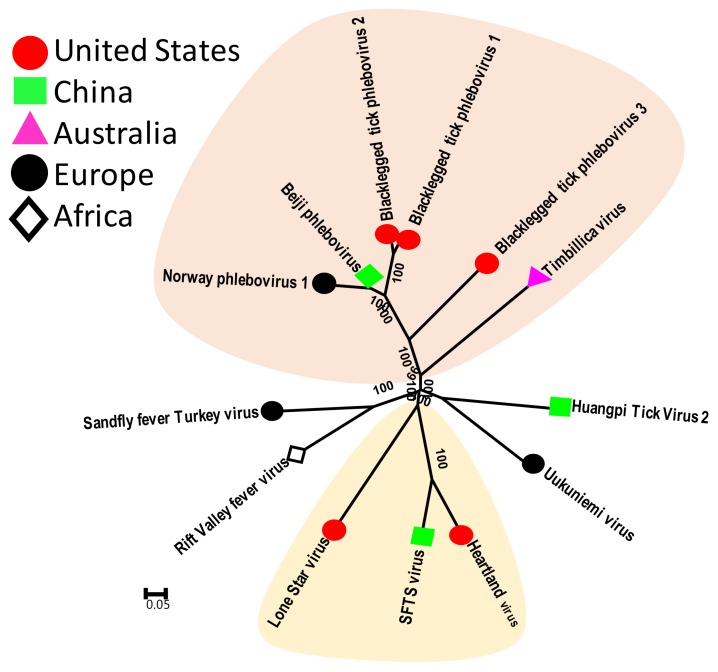
Phylogenetic relatedness among tick phleboviruses. This analysis involved 13 amino acid sequences of the viral polymerase gene. Genbank accession numbers of sequences used for phylogenetic analysis are: YP_009293590 Huangpi Tick Virus 2, NP_941973 Uukuniemi virus, YP_004382743 Sandfly fever Turkey virus, YP_003848704 Rift Valley fever virus, YP_006504091 SFTS virus HB29, YP_009047242 Heartland virus, ANC97695 Black-legged tick phlebovirus 3, ASY03242 Norway phlebovirus 1, AII01803 Black-legged tick phlebovirus 2, ANT80544 Black-legged tick phlebovirus 1, AYP67564 Timbillica virus, AXQ59272 Beiji phlebovirus and YP_008003507 Lone Star virus.

These viruses belong to the family *Phenuiviridae* of the order *Bunyavirales*. Tick phleboviruses include the well-characterized SFTSV and Heartland Virus, and several recently identified viruses such as Black-legged tick phlebovirus type 1, 2 and 3 (United States), Norway Phlebovirus 1, American dog tick phlebovirus, Beiji Phlebovirus (China) and Timbillica virus (Australia) (Figure 2). Phleboviruses resemble nairoviruses in genomic organization and, as described earlier for nairoviruses, only two of the three genomic segments of tick phleboviruses have been identified so far [17,18,39]. Three new genotypes of tick phleboviruses were identified form *I. scapularis* and one new genotype was identified from *D. variabilis* from the United States. These viruses were henceforth named Black-legged tick Phlebovirus 1–3, and American dog tick phlebovirus, respectively. All of these viruses were found in >50% of adult ticks, indicating they are highly prevalent infections [17,18,39]. Similarly, the studies of *I. ricinus* ticks from Norway, *I. persulcatus* from China and *I. holocyclus* from Australia all showed the presence of new phleboviruses that were tentatively named Norway phlebovirus 1, Beiji phlebovirus and Timblica virus, respectively [38,40]. Comparative phylogenetic analysis indicates that these new tick phleboviruses are highly divergent and they form a unique genetic clade that is equally different from all known tick phleboviruses, such as Heartland Virus and SFTSV.

#### 3.2.3. Tick Chuviruses 

**Figure 3:**
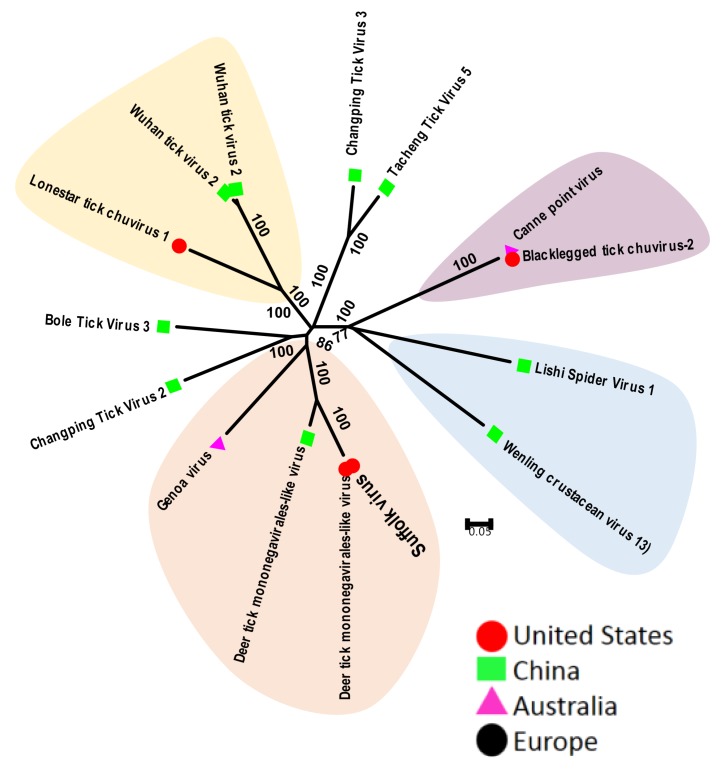
Phylogenetic relatedness between tick chuviruses. This analysis used 15 amino acid sequences of the viral polymerase gene. Genbank accession numbers of sequences used for phylogenetic analysis are: YP_009177218 Suffolk virus, AIE42676 Deer tick mononegavirales-like virus, AXQ59273 Deer tick mononegavirales-like virus, AYP67566 Genoa virus, YP_009177704 Changping Tick Virus 2, YP_009177701 Bole Tick Virus 3, YP_009254000 Lonestar tick chuvirus 1, YP_009177707 Changping Tick Virus 3, YP_009177717 Tacheng Tick Virus 5, YP_009177722 Wuhan tick virus 2, AYV61060 Wuhan tick virus 2, AYP67535 Canne point virus, AJG39051 Lishi Spider Virus 1, AUW34382.1 L Black-legged tick chuvirus-2 and YP_009337860 Wenling crustacean virus 13.

These viruses belong to the newly created family *Chuvirus* of the order *Mononegavirales*. These viruses have linear or circular genomes that consist of a single-strand negative-sense RNA that codes for all viral proteins. Tick mononegavirus was first identified in *I. scapularis* [17]. The complete genome of this virus remained unknown until the identification of this new group of arthropod viruses, called chuviruses [44]. Several genetically related chuviruses have been identified from *I. scapularis* and *A. americanum* ticks from several states within the United States [17]. Tick chuviruses include Suffolk virus, Lonestar tick chuvirus and Black-legged tick chuvirus from the United States, Deer tick mononegavirales-like virus, Changping Tick virus 2 and 3, Bole tick virus 3, Wuhan tick virus 2 and 3, Tacheng tick virus 5 from China, and Genoa virus and Canne point virus from Australia. Tick chuviruses are highly prevalent and can be found in multiple tick species in the same geographic regions [39]. Comparative phylogenetic analysis of these viruses indicates that viruses from different species of ticks are more divergent, even if these ticks were collected from same country. Similarly, viruses from related species of ticks, such as Suffolk, Genoa and Deer tick mononegavirale-like virus from China were isolated from *Ixodes* spp. and were genetically clustered (Figure 3). Finally, the Black-legged tick chuviruses from the United States and Canne point virus from Australia were almost identical, while both were the most divergent from all of the other tick chuviruses; they clustered more closely with the chuviruses found in crustaceans and spiders.

#### 3.2.4. Tick Flaviviruses

The most commonly found members of family *Flaviviridae* were Powassan or Deer tick virus and Jingmen-tick virus, but the study from Australia reported the presence of hepaciviruses and pestiviruses [37], that if confirmed would shed new light on the origin and transmission of these viruses. Interestingly, a study from Europe also reported the presence of Dengue, Hepatitis C and GB virus-B such as sequences from ticks, but all these sequence reads were short and therefore require confirmation [40]. Furthermore, metagenomic data are often contaminated with viruses from the environment and ingested or digested food [42].

**Figure 4:**
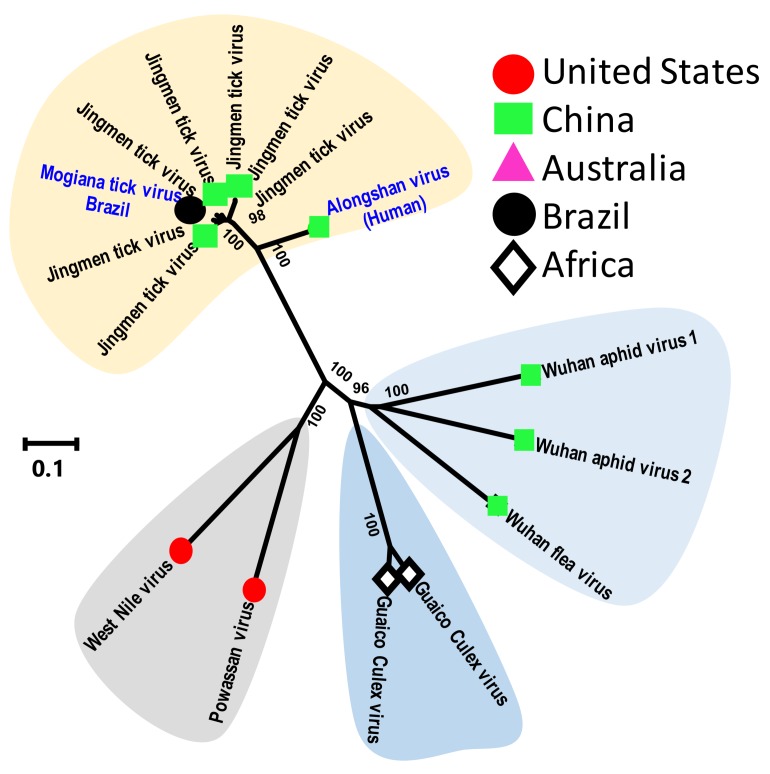
Phylogenetic relatedness based on the NS3-helicase protein of the JMTV-like viruses of ticks, aphids and mosquitoes and classical flaviviruses (Powassan and West Nile virus). This analysis involved 16 amino acid sequences. All ambiguous positions were removed for each sequence pair. There were a total of 983 positions in the final dataset. Genbank accession numbers of sequences used for phylogenetic analysis are: YP_009030000 Jingmen tick virus, YP_009351918 Mogiana tick virus, YP_009179379 Wuhan aphid virus 2, YP_009179404 Wuhan flea virus, YP_009179389 Wuhan aphid virus 1, AYV61015 Jingmen tick virus, AYV61032 Jingmen tick virus, AYV61020 Jingmen tick virus, AXH38008 Jingmen tick virus, AXH38007 Jingmen tick virus, AXE71876 Alongshan virus, AHZ31717 Jingmen tick virus, ALP82430 Powassan virus, AKL90418 Guaico Culex virus, AKL90453 Guaico Culex virus and AFI56962 West Nile virus.

The most interesting of the new tick viruses is Jingmen tick virus (JMTV), which is also the first well-characterized segmented flavivirus [43]. The JMTV genome contains four single-strand positive-sense RNA segments that encode a flavivirus-like helicase, a polymerase and structural proteins. This virus was first identified in China from *Rhipicephalus microplus* ticks [43]. Interestingly, PCR-based surveillance indicates that JMTV infection has been found in eight different species of ticks; *R. microplus* (114 of 181), *H. longicornis* (50 of 91), and *H. campanulata* (18 of 24) had the highest positive rates, followed by *H. flava* (5 of 45). The remaining tick species—including *R. sanguineus* (1 of 3), *I. sinensis* (1 of 1), and *I. granulatus* (1 of 3)—all contained JMTV-positive samples. Evidence of JMTV infection was also reported from a wide range of rodents and cattle. Although from an unpublished report, a JMTV-related virus (Alongshan virus GenBank no. AXE7876.1) was reported to be found in humans with febrile illness. Another relative of JMTV, Mogiana tick virus was recently found in *R. microplus* ticks from Brazil [49]. Finally, genetically related viruses are also reported from mosquitoes and were shown to be capable of infecting mammalian hosts [50]. Comparative phylogenetic analysis of JMTV-related viruses from ticks and mosquitoes indicate that they form two distinct genetic clusters, with the mosquito viruses are more similar to aphid viruses (Figure 4). Most importantly, these different studies suggest that JMTV and genetically related viruses possess strong potential for cross-species transmission from ticks to mammals, including primates (and humans) [43,49,50,51], and thus warrant urgent investigations into their origin, ecology and host tropism.

## 4. Summary and Future Directions

Undoubtedly, our knowledge of the viruses present in ticks has been substantially increased in the last few years, but a deep dive into data generated by metagenomics indicates that most of the studies designed to characterize the tick virome used pools of ticks as study samples and, thus, it is plausible that rare or less abundant tick viruses have eluded identification. This hypothesis is further supported by the observation that, of several well-known tick viruses, only a few have been found using metagenomics. Two ways to improve the analysis of the tick virome include using the metagenomics of large numbers of individual ticks and/or the enrichment of samples for viruses that are more likely to infect mammalian cells. Tick homogenates can be inoculated in cell lines or animal models to enrich viruses with the potential to infect vertebrate hosts, followed by metaviromic-based identification.

Another important issue is our limited knowledge of the origin and ecology of these newly discovered tick viruses. The tick virome is comprised of innate and/or acquired viruses. Innate viruses can be endogenous viruses, those integrated in the genome, and viruses that are exclusively present in ticks and maintained through vertical transmission. So far, a thorough analysis of tick eggs and larvae has not been reported and this could shed some light on the nature of innate virome of ticks. Similarly, only a few acquired tick viruses have been characterized for their transmission and ecology. The studies of these viruses, such as Powassan or CCHFV, indicate that these viruses are acquired by ticks from mammalian reservoir hosts during the blood meals associated with the tick lifecycle. These viruses then remain within the tick and vertebrate host cycle until they incidentally spill over and infect humans. Interestingly, the complete host range of these viruses is still not fully understood since most of these viruses cause a transient viremia in the vertebrate host that may or may not be detected using PCR-based assays. We believe the development of serological assays for tick viruses will substantially improve our knowledge of virus ecology and host tropism. Serological assays can also reveal the real infection prevalence of known or new tick viruses in vertebrate hosts, since the presence of antibodies can indicate both prior and ongoing viral infection.

Lastly, it is important to surveil domestic, feral and wild animals for both the presence of tick viruses and evidence of viral infections. It is plausible that the ongoing transmission of tick viruses in synanthropic animals is providing tick viruses an interface to evolve and establish infection in humans. Infection with Powassan virus in a range of wild rodents and other mammals is well documented and this supports the maintenance and evolution of tick viruses in synanthropic hosts [52]. The identification of host ranges and a better understanding of the community ecology of these tick viruses will not only be very helpful in preventing their transmission to humans, but will also identify animal species that can be subsequently used to develop informative animal models for the studies of both novel and known tick viruses.

## Figures and Tables

**Table 1:** At-a-glance view of the global tick virome.

Virome	Tick Species	Virus Names	References
**United States**	*Ixodes scapularis*	*South Bay virus, Suffolk virus, Phleboviruses, Rhabdoviruses, Powassan virus*	[17,18,39]
	*Dermacentor variabilis*	*Phlebovirus, Rhabdoviruses, Noda or Tetravirus-like virus*	
	*Amblyomma americanum*	*Rhabdovirus*	
**Norway (Europe)**	*Ixodes ricinus*	*Phleboviruses, Nairovirus, Churivirus, Luteovirus*	[38]
**France (Europe)**	*Ixodes ricinus*	*Nairovirus*, *Phlebovirus*, *Coltivirus (Eyach virus)*, *Rhabdoviridae*	[40]
**Australia**	*Amblyomma moreliae, Ixodes trichosuri* and *Ixodes holocyclus*	*Chuviridae, Flaviviridae, Luteoviridae, Narnaviridae, Orthomyxoviridae, Partitiviridae, Phenuiviridae,, Picornaviridae, Reoviridae, Rhabdoviridae, Unclassified−Mononegavirales, Virgaviridae*	[37]
**China**	*Ixodes persulcatus, Dermacentor nuttalli, Dermacentor silvarum, Haemaphysalis longicornis*, and *Haemaphysalis concinna.*	*Phleboviruses, Nairovirus, Churivirus* and *Jingmen tick virus*	[36]
	*Rhipicephalus* spp.	*Nairovirus, Rhabdovirus*	[41]
